# PReS-FINAL-2199: A novel mutation in the CIAS1/NLRP3 gene associated with an unusual phenotype of CAPS

**DOI:** 10.1186/1546-0096-11-S2-P189

**Published:** 2013-12-05

**Authors:** A Insalaco, G Prencipe, PS Buonuomo, I Ceccherini, C Bracaglia, M Pardeo, R Nicolai, F De Benedetti

**Affiliations:** 1 Pediatric Medicine, Rheumatology, Ospedale Pediatrico Bambino Gesù, Rome, Italy; 2 Molecular Genetic Laboratory, Istituto Giannina Gaslini, Genoa, Italy

## Introduction

Cryopyrin-associated periodic syndrome (CAPS) is an autoinflammatory syndrome caused by heterozygous mutations of CIAS1/NLRP3 gene. Affected patients may present three different phenotypes: familial cold autoinflammatory syndrome (FCAS), Muckle-Wells syndrome and CINCA syndrome. Common symptoms include sporadic or cold-induced non pruritic urticarial rash and fever. Severe cases suffers from deafness, meningitis, articular contracture and secondary amyloidosis.

## Objectives

To describe a patients with a novel mutation associated with an unexpected phenotype.

## Methods

We describe a 13-year-old female who presented, starting at 12 years of age, recurrent episodes of high fever, pericarditis, arthralgia, arthritis of the knees, abdominal pain and marked increase in inflammatory markers (5 episodes in the first 5 months of disease). Symptoms were poorly responsive to therapy with NSAIDs and colchicine but responded to steroid therapy. Molecular analysis of MEFV, TNFR and MVK genes did not show any pathogenic mutations. Serum amyloid level was elevated. In the subsequent months she developed recurrent (up to daily) episodes of chest pain, skin rash and swelling of the subcutaneous tissue of limbs, trunk, joints and lips, in the absence of fever, with spontaneous resolution.

## Results

Molecular analysis of the CIAS1 gene revealed the presence of a c.1105C>A mutation in the heterozygous state, that predicts a L369M amino acid substitution. To the best of our knowledge this variant has never been reported. One-Hundred chromosomes were examined and the variant was not found. In the same exon, a heterozygous c.2107C>A nucleotide substitution, leading to the known p.Q703K amino acid substitution has also been detected.

We then evaluate the potential relevance of this mutation on the deregulation of il1b production. Plasma IL-1b levels were elevated compared to healthy controls. More importantly, in vitro IL-1b production by peripheral blood mononuclear cells, was increased; in particular, stimulation with LPS alone induced detectable IL-1b in our patient in contrast to controls. In order to verify the potential in vivo role of the observed L369M amino acid substitution, daily therapy with anakinra (2 mg/Kg/day) was started with rapid disappearance of clinical symptoms and normalization of CRP levels in 24 hours.

## Conclusion

The rapid and complete response to IL-1 inhibition suggests that the clinical and laboratory features of the disease of this patient are driven by IL-1 Our data in silico, in vitro and in vivo support the conclusion that this novel mutation is pathogenic and may be associated with a new CAPS phenotype. The role played by the concomitant presence of the Q703K variant remains to be clarified, though an effect in modifying the disease features cannot be excluded.

## Disclosure of interest

None declared.

